# Intensity distribution segmentation in ultrafast Doppler combined with scanning laser confocal microscopy for assessing vascular changes associated with ageing in murine hippocampi

**DOI:** 10.1038/s41598-022-10457-9

**Published:** 2022-04-26

**Authors:** Maximiliano Anzibar Fialho, Lucia Vázquez Alberdi, Mariana Martínez, Miguel Calero, Jerome Baranger, Mickael Tanter, Juan Pablo Damián, Carlos Negreira, Nicolás Rubido, Alejandra Kun, Javier Brum

**Affiliations:** 1 Laboratorio de Acústica Ultrasonora, Instituto de Física, Facultad de Ciencias, Universidad de la República, 11400 Montevideo, Uruguay; 2 Física No Lineal, Instituto de Física de Facultad de Ciencias, Universidad de la República, 11400 Montevideo, Uruguay; 3 Laboratorio de Biología Celular del Sistema Nervioso Periférico, Departamento de Proteínas y Ácidos Nucleicos, Instituto de Investigaciones Biológicas Clemente Estable, 11600 Montevideo, Uruguay; 4 Chronic Disease Programme (UFIEC), Instituto de Salud Carlos III, CIBERNED and CIEN Foundation, 28220 Madrid, Spain; 5 Physics for Medicine Paris, Inserm U1273, ESPCI Paris, PSL University, CNRS UMR 8063, 75012 Paris, France; 6 Departamento de Biociencias Veterinarias, Facultad de Veterinaria, Universidad de la República, 13000 Montevideo, Uruguay; 7 Present Address: Institute for Complex Systems and Mathematical Biology, University of Aberdeen, King’s College, Aberdeen, AB24 3UE UK; 8 Sección Bioquímica, Facultad de Ciencias, Universidad de la República, 11400 Montevideo, Uruguay

**Keywords:** Confocal microscopy, Ultrasound, Neural ageing, Imaging techniques, Ageing

## Abstract

The hippocampus plays an important role in learning and memory, requiring high-neuronal oxygenation. Understanding the relationship between blood flow and vascular structure—and how it changes with ageing—is physiologically and anatomically relevant. Ultrafast Doppler ($\mu$Doppler) and scanning laser confocal microscopy (SLCM) are powerful imaging modalities that can measure in vivo cerebral blood volume (CBV) and post mortem vascular structure, respectively. Here, we apply both imaging modalities to a cross-sectional and longitudinal study of hippocampi vasculature in wild-type mice brains. We introduce a segmentation of CBV distribution obtained from $\mu$Doppler and show that this mice-independent and mesoscopic measurement is correlated with vessel volume fraction (VVF) distribution obtained from SLCM—e.g., high CBV relates to specific vessel locations with large VVF. Moreover, we find significant changes in CBV distribution and vasculature due to ageing (5 vs. 21 month-old mice), highlighting the sensitivity of our approach. Overall, we are able to associate CBV with vascular structure—and track its longitudinal changes—at the artery-vein, venules, arteriole, and capillary levels. We believe that this combined approach can be a powerful tool for studying other acute (e.g., brain injuries), progressive (e.g., neurodegeneration) or induced pathological changes.

## Introduction

Brain homeostasis results from a fine balance between brain’s perfusion and metabolism, whereby nutrient/oxygen supply is provided by the blood flow in response to a complex neuro-glial-pericyte-vascular system. With ageing, subtle changes progressively alter the neurovascular coupling, due to both endothelial dysfunction, resulting in a regional decrease in the vascular reserve capacity, and cellular changes in oxidative stress levels and increased inflammation^1–3^. Acute or chronic alterations in cerebral blood flow compromise oxygen or glucose supply and can affect memory, cognitive and executive functions, as has been reported for several nervous system diseases^4–7^.

A specially relevant brain region is the hippocampus, due to the important cognitive processes it supports and its exclusive neurogenic capacity in mammals^1,8–10^. It is also one of the most affected areas in Alzheimer’s disease. Recently, it has been shown that there is a physiopathological relationship between vascular alterations and the onset of the Alzheimer’s disease^11–14^.

Ultrafast Doppler ($\mu$Doppler)^15^ has proven to be a powerful tool for in vivo blood flow imaging of the brain. It provides highly sensitive imaging of cerebral blood volume (CBV) by merging power Doppler^16^ and ultrasound ultrafast imaging^17^. $\mu$Doppler uses successive ultrasonic tilted plane-wave emissions acquired at ultrafast frame rates (up to 20 kHz) which are coherently compounded and accumulated. Combined with optimized spatiotemporal filters for discrimination between tissue and blood flow motion, this accumulation step enables a sensitivity increase up to a factor of 50 in the signal-to-noise ratio when compared to conventional power Doppler with line-by-line scan^18^. This sensitivity boost allows imaging of low blood flow speeds (down to 1 mm/s) associated with small arterioles in the brain with a spatial resolution of 100–200 $\upmu$m depending on the ultrasonic frequency used^18^. However, $\mu$Doppler imaging of the brain has mostly been conducted for functional-ultrasound neuroimaging purposes, and data about the relationship between $\mu$Doppler’s CBV and the underlying vascular structure are scarce.

Confocal laser scanning microscopy (SLCM) is an exquisite optical microscopy modality, which is based on specimen point lighting associated to the elimination of unfocused light from other focal planes, with optical sectioning system and 3D reconstructions. With a spatial resolution between 225 to 250 nm (or even higher in super-resolution SLCM), it is mainly used for in situ molecular analysis of biological samples, fixed or in vivo^19^. The resultant image gives faithful molecular interrelationships, cell structures or tissue/organ organization^20,21^. Particularly, this allows characterizing vascular tissular network at different levels, such as artery-vein, venules, arterioles and capillary^22–26^. However, preserving the structure of cerebral vascular network and their molecular interrelationships with a high-resolution level, usually implies brain fixation during or immediately after death to avoid deterioration by anoxia effects.

In this work, we study the relationship between CBV and vascular structure in 12 wild type (WT) mice of 5 and 21 months old at the hippocampal level by $\mu$Doppler and SLCM imaging modalities. We analyse the experimental data by implementing a combined approach between in vivo $\mu$Doppler and high-resolution post mortem SLCM, instead of trying to increase $\mu$Doppler’s resolution^27,28^. We quantify CBV in the hippocampus by segmenting each $\mu$Doppler image in quartiles of their intensity distribution, making the quartile cut-off values a mice-independent measure that can be used in robust inter-cohort statistical analysis. Subsequently, we characterize vascular structure by SLCM in the same area of interest, obtaining number of vessels and vessel volume distributions that we also segment into ranges, aligned with the reserve capacity of vein-artery, venule, arteriole, and capillary levels^29–31^. Our results show that high CBV is related to specific vessel locations with large volume fractions. Moreover, we find significant changes appearing in the CBV distribution and vasculature due to ageing.

## Methods

### Animal preparation

All animal experiments and procedures were approved by the local ethics committee (Comisión de Ética en el Uso de Animales (CEUA), Instituto de Investigaciones Biológicas Clemente Estable (IIBCE), Uruguay, protocol number: 002a/10/2020). All experiments were carried out in strict accordance with the relevant regulations and guidelines (Uruguayan law number 18611). The study is reported in accordance with ARRIVE guidelines. Wild type C57BL/6 mice were obtained from Jackson Laboratories. The colony was raised at the IIBCE animal house in a controlled environment (12 h. dark/12 h. light cycle, average temperature of 21 ±  3 $^\circ$C), with unrestricted access to food and water. At 21 days of age the mice were weaned, sexed and numbered by ear punching method. Twelve male mice of 5-month-old (n = 6) and 21-month-old (n = 6) were used in the $\mu$Doppler experiments. Of these twelve mice, ten were used for vascular structure evaluation using confocal microscopy imaging (five 5-month-old and five 21-month-old, respectively).

**Figure 1:**
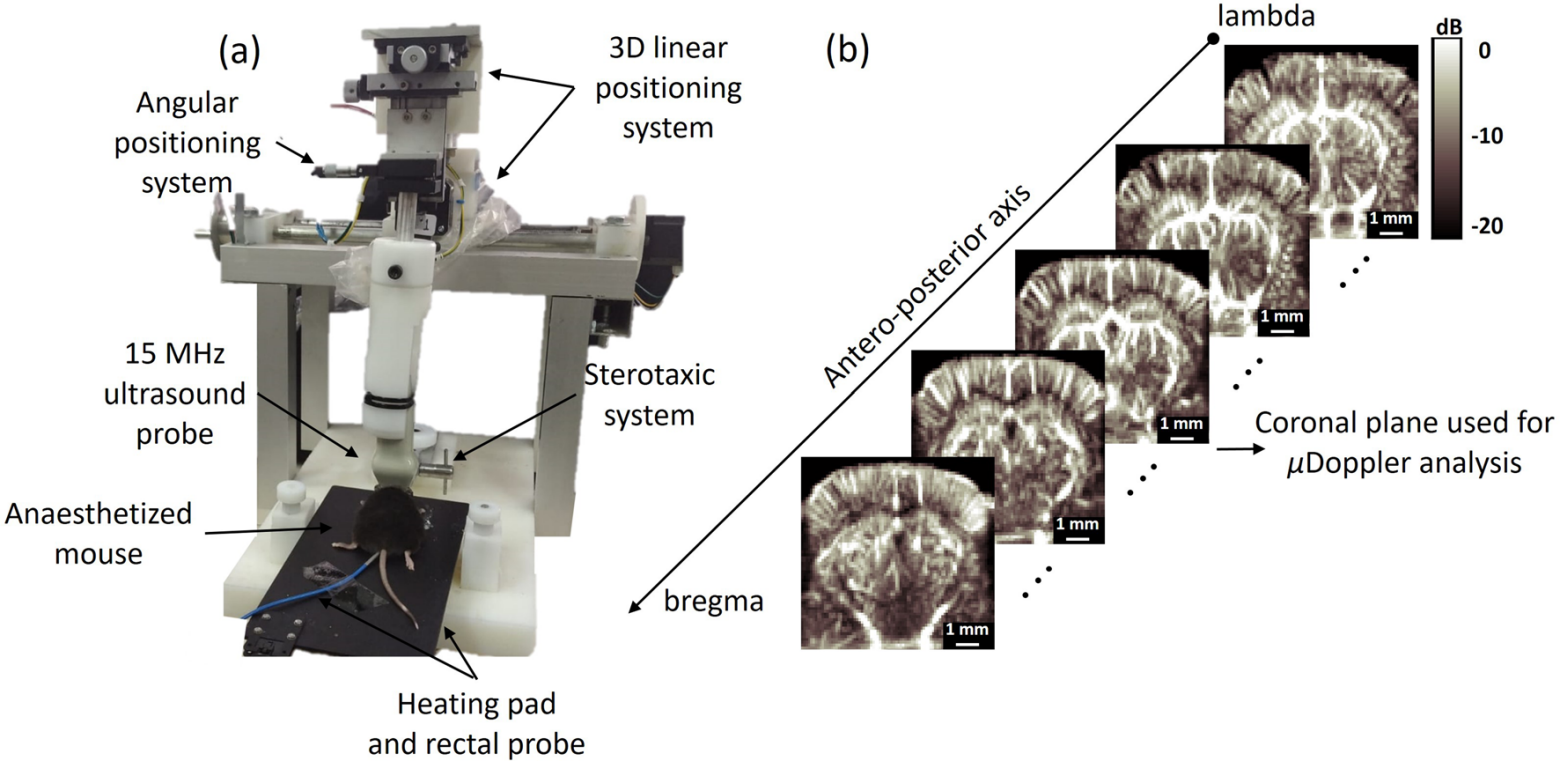
Experimental set-up used in $\mu$Doppler experiments. (**a**) After craniotomy, the anaesthetized mouse was placed in a stereotaxic system. A 15 MHz ultrasound probe driven by a Verasonics system was positioned over the cranial window and aligned to the coronal plane of the brain. Then, the probe was moved along the anteroposterior axis to image the whole brain using a 3D linear positioning system driven by a step-by-step motor. (**b**) In this example we show different coronal planes acquired for a 5-month-old mouse. In this experiment the whole scan consisted of 32 planes separated by 0.1 mm. For the sake of clarity some planes were omitted in (**b**).

Anaesthesia was prepared by dissolving 120 mg/kg ketamine (Vetanarcol, König) and 16 mg/kg xylazine (Xylased*2, Vetcross) in saline solution to a final volume of 300 $\upmu$l. For the experiments, mice were anesthetized with one half of this solution (150 $\upmu$l) through intraperitoneal injection, while observing the animal’s reaction. If necessary, the rest of the solution was injected. Once the mice were anesthetized, a $4 \times 6$ mm$^2$ cranial window was surgically opened in order to expose the brain and to allow undistored propagation of ultrasound. Next, mice were positioned in a stereotaxic frame, fixing the mouse’s head while conducting $\mu$Doppler. Figure 1a presents the experimental set-up used in the $\mu$Doppler experiments. The mouse temperature was held at 37 $^\circ$C using a rectal probe (HP-1M thermocouple, Physitemp, USA) and a heating pad (HP-1M, Physitemp, USA) both connected to a temperature controller (TCAT-2DF, Physitemp, USA).

### CBV imaging and analysis

#### $\mu$Doppler ultrasonic sequence

A 128 element, 15 MHz probe (Vermon) driven by Verasonics Vantage System was aligned to the coronal plane of the brain. The probe was moved along the anteroposterior axis by a step-by-step motor (0.1 mm step) to scan the whole cranial window (Fig. 1b). Each $\mu$Doppler image was built from averaging 350 compound images. Tilted plane wave emission from four angles (− 6$^\circ$, − 2$^\circ$, 2$^\circ$ and 6$^\circ$) were added coherently to form a compound image^32,33^. To increase the signal-to-noise ratio, each tilted plane wave was emitted three times and its backscattered echoes were automatically averaged in the Verasonics Vantage System memory (i.e. memory accumulation was set to three). All emission/reception times were adjusted to achieve a $\mu$Doppler frame rate of 500 Hz. The lateral (i.e. probe’s pitch) and axial pixel resolution were 0.1 mm. The post-compounding dataset was arranged in an $N_x$ $\times$ $N_z$ $\times$ $N_t$ matrix with $N_x = 128$ (i.e. element number of the probe), $N_z = 92$ and $N_t = 350$ (number of compounded images).

#### Clutter filtering

After ultrasonic acquisition, a clutter filter based on Singular Value Decomposition (SVD)^34,35^ was used to discriminate between tissue, blood and noise in the ultrasonic signals. To this end, the data was reshaped to a 2D Casorati matrix S with dimensions $N_x.N_z$ by $N_t$. After SVD the matrix S was written as S = UDV*, where D is an ($N_x.N_z$, $N_t$) diagonal matrix with diagonal coefficients $\lambda _i$ (sorted in descending order) and matrices U and V are unitary matrices containing the singular vectors for each singular value $\lambda _i$. Tissue signal (with high energy and spatial coherence) is associated with the first singular values while noise signal (with low energy, spatially and temporally incoherent) will be concentrated on the last singular values^35^. The clutter filter consists of suppressing tissue and noise by using a filtering matrix F that removes the contribution of the first and last singular values from S. The matrix F is diagonal, with ones for the elements between $N_{tissue}$ and $N_{noise}$ and zero for the rest. $N_{tissue}$ and $N_{noise}$ are the numbers corresponding to the low and high order singular value cut-off thresholds used to reject tissue and noise, respectively. Therefore, the filtered matrix $S_{blood}$ associated with blood signal is written as $S_{blood}$ = U(D.F)V*.

In this work, the optimal threshold values $N_{tissue}$ and $N_{noise}$ were chosen to maximize the signal-to-noise ratio (SNR) from blood with respect to tissue and noise. The SNR was computed following a similar procedure to^28,36^

1 $$\begin{aligned} SNR=\frac{{{\overline{S}}}_{blood}}{{{\overline{S}}}_{noise/tissue}} \end{aligned}$$

where ${{\overline{S}}}_{blood}$ is the average blood signal within a region of interest (ROI) inside the hippocampus containing at least one vessel and ${{\overline{S}}}_{noise/tissue}$ is the average signal associated with noise and tissue computed for a ROI outside the hippocampus with no visible vessels. The ROI’s size was of 0.5 $\times$ 0.5 $\hbox {mm}^2$ and its location was approximately the same for all mice. The ROI for blood was located on the inter-hemispheric fissure, while the ROI for noise was chosen inside the central ventricle where no Doppler signal is expected due to the absence of vessels. Mean threshold values of $N_{tissue}$ = 30 ± 11 and $N_{noise}$ = 78 ± 14 (mean value ± standard deviation ) were found for all mice. This low variation of the threshold values indicates that clutter conditions (e.g. tissue motion) and flow ranges were comparable among different mice^35^.

**Figure 2:**
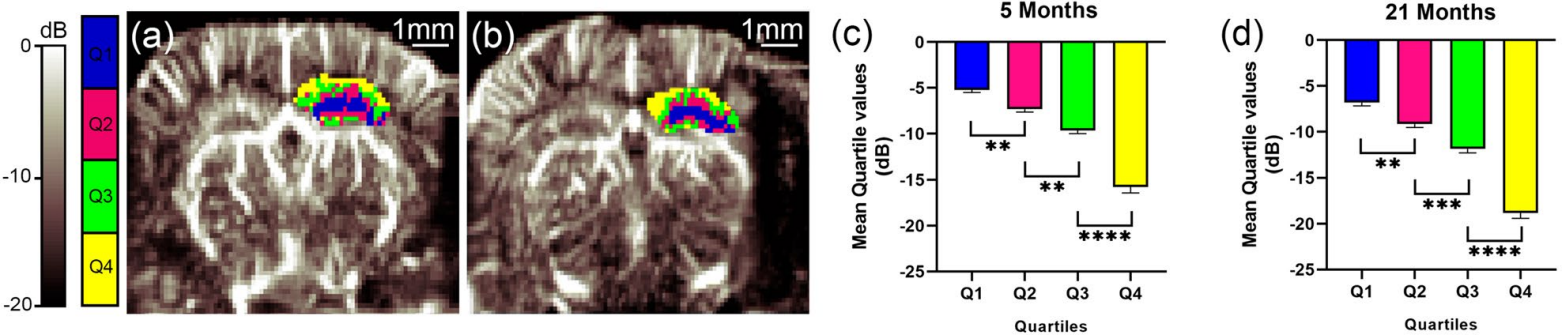
$\mu$Doppler segmentation of the hippocampus. Coronal $\mu$Doppler image in decibels (dB) of a (**a**) 5-month-old and (**b**) 21-month-old mouse. For the dB scale, the reference value was taken as the maximum intensity within left hippocampus. The right hippocampus is overlaid with the quartile distribution of the flow intensity. Blue, fuchsia, green and yellow corresponds to pixels within the quartiles Q1, Q2, Q3 and Q4, respectively. The mean quartiles values showed significant differences between all quartiles for (**c**) 5-month-old and (**d**) 21-month-old mice. For 5-months-old mice: $\hbox {F}_{(3,44)} = 3{.}911$; [Q1 vs Q2]: p = 0.0056, [Q2 vs Q3]: p = 0.0019 and [Q3 vs Q4]: p < 0.0001. For 21-months-old mice, $\hbox {F}_{(3,44)}$ = 1.806; [Q1 vs Q2]: p = 0.0029, [Q2 vs Q3]: p = 0.0005, [Q3 vs Q4]: p < 0.0001. **p < 0.0021, ***p < 0.0002, ****p < 0.0001.

#### $\mu$Doppler analysis in the hippocampus

The final image is the result of an average of twenty $\mu$Doppler images. For the analysis, one image corresponding to approximately bregma − 1.58 mm was used (Fig. 1b). An example of $\mu$Doppler images is shown in Figs. 2a,b for a 5 and 21 month-old mice, respectively. For each image, the region occupied by the hippocampus was manually selected by comparing the $\mu$Doppler image to the Praxinos and Franklin’s mouse brain atlas^37^ along with the corresponding SLCM image. Then, the $\mu$Doppler signal within the hippocampus was converted into decibels (dB) and segmented using the quartile cut-off values (Q1—25$\%$, Q2—50$\%$, Q3—75$\%$ and Q4—100$\%$) of their intensity distribution (Fig. 2). For the dB conversion, the reference value was taken as the maximum intensity within the hippocampus. Left and right hippocampi were treated independently. In Fig. 2a,b the right hippocampus after quartile segmentation is highlighted in color.

### Vascular structure imaging and processing

#### Brain preparation and vibratome sectioning

After $\mu$Doppler imaging, mice were euthanized with a quick cervical dislocation^38^. The brain samples were processed as described by Damián et al.^39^. Briefly, after cervical dislocation, brains were immersed in 4$\%$ paraformaldehyde (PFA) fixative solution in PHEM buffer (60 mM PIPES, 25 mM HEPES, 10 mM EGTA, 2 mM $\hbox {MgCl}_2$, adjusted to pH 7.2–7.4 with KOH pellets) for 1 h. at 4 $^\circ$C in an orbital shaker. Brains were stored at 4 $^\circ$C for 24 h. in a freshly prepared 4$\%$ PFA. Finally, the PFA leftovers were eliminated by washing the brain in PHEM buffer using an orbital shaker. After fixation, the brains were immediately included in a support of 4$\%$ agarose in water (modified from Moreno-Jiménez et al.^10^), and 50 $\upmu$m contiguous vibratome thick sections were obtained (Leica, VT 1000S). All brain slices were stored in PHEM solution at 4 $^\circ$C until used for vascular structure recognition by SLCM.

#### Vascular recognition with IB4 fluorescent probe

Brain slices were incubated with an IB4 fluorescent probe (Isolectin GS-IB4 Alexa Fluor 488 Conjugate, Thermofisher) in 1:100 concentration with PHM buffer (60 mM PIPES, 25 mM HEPES, 2 mM $\hbox {MgCl}_2$, adjusted to pH 7.2–7.4 with KOH pellets) and 0.5 mM $\hbox {CaCl}_2$. Brain slices were incubated over night at 4 $^\circ$C and washed for 5 min with stirring (X6). During incubation, isolectin agglutinates with perivascular cells, probing the vessel wall with a green fluorescent spectrum. Finally, brain slices were mounted in a slide with Prolong Antifade Diamond (ThermoFisher) and were allowed to dry in the dark for 24 h before SLCM imaging.

#### Confocal imaging

**Figure 3:**
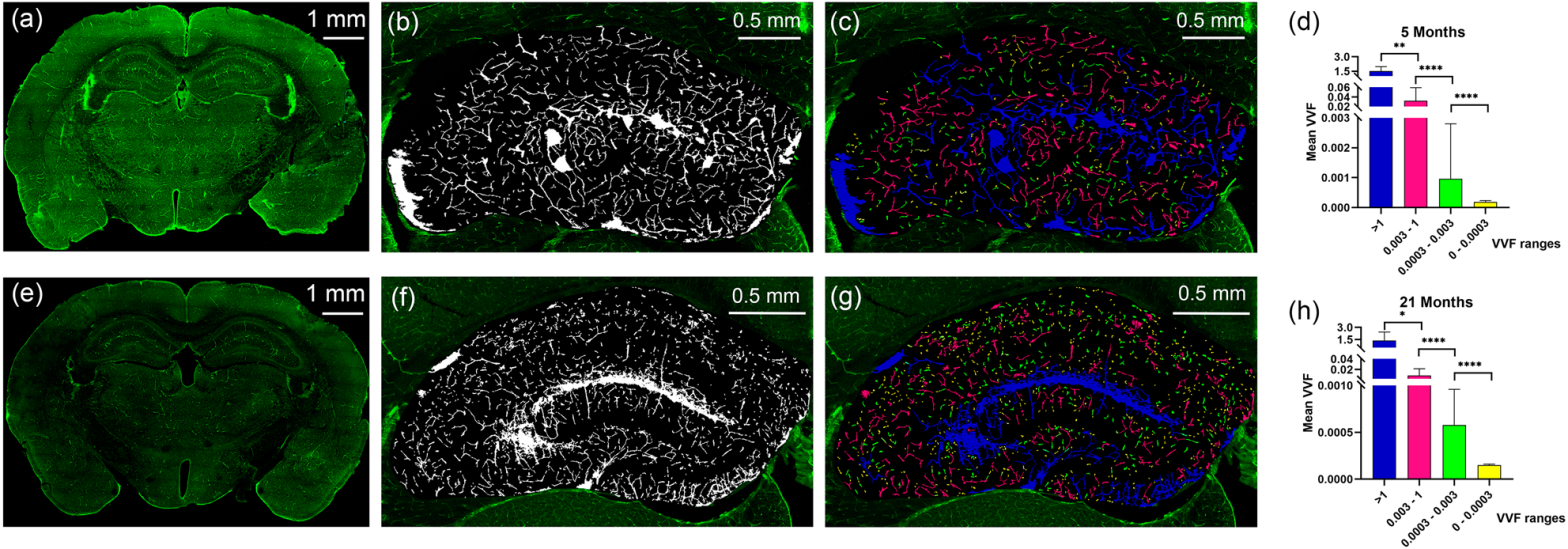
Vascular structure in the hippocampus analyzed by SLCM. (**a**) Tile-scan image of a coronal section for a 5-month-old mouse. The vascular endothelium marked with IB4 appears green. (**b**) Binary image in grey scale computed by the ImageJ automatic threshold function from the hippocampus in (**a**). (**c**) 3D counter object image showing the distribution of identified vessels. Vessels in the 0–0.0003, 0.0003–0.003, 0.003–1 and > 1 range were coloured yellow, green, fuchsia and blue, respectively. (**e**)–(**g**) Same as (**a**)–(**c**) but for a 21-month-old mouse. (**d**) and (**h**) show the mean vessel volume fraction (VVF) for all 5-month-old and 21-month-old mice included in this study, respectively. Significant differences were found for all ranges. For 5-month-old mice: $\chi ^2$(4) = 30; [0–0.0003 vs. 0.0003–0.003] and [0.0003–0.003 vs. 0.003–1]: p < 0.0001, [0.003–1 vs. > 1]: p = 0.0006. For 21 month old mice: $\chi ^2$(4) = 30; [0–0.0003 vs. 0.0003–0.003] and [0.0003–0.003 vs. 0.003–1]: p < 0.0001, [0.003–1 vs. > 1]: p = 0.0440. *p < 0.05, **p < 0.0021, ****p < 0.0001.

IB4 fluorescence images of each brain slice were obtained using a Zeiss LSM 800 confocal microscope with an airy scan module. First, the specific photomultiplier laser maximal levels were fixed with the negative controls of each sample, using mode levels of saturation, until a few brilliant non-specific signals started to appear. Then, all the samples containing IB4 were taken under the same conditions, using the same SLCM section. The voltage values of the photomultipliers never exceeded the initial ones set with the control samples, and they were lowered when fluorescence intensity saturation appeared. This procedure ensured equal conditions for fluorescence intensity quantification. The final SLCM image is composed of 10 adjacent planes forming a z-stack. The distance between planes (z-step) was set to 5 $\upmu$m. Images were acquired with 10$\times$ (pixel size 0.56 $\upmu$m) and 20$\times$ (pixel size 0.28 $\upmu$m) panoramic lenses using the tail-scan mode with a three-axis motorized stage to cover the entire coronal section of the brain (Fig. 3a,e).

#### Image analysis

For the analysis, two confocal images corresponding to consecutive brain slices of $\approx$ 50 $\upmu$m were used. This $\approx$ 100 $\upmu$m thickness is similar to the $\mu$Doppler ultrasound beam width in elevation. Each confocal image was imported into ImageJ software (version 1.53b, RRID: SCR_003070) for fluorescent intensity analysis. To this end, each hippocampus was selected from the acquired image of each coronal section. For each z-stack plane a binary image was created by using the ImageJ automatic threshold function (Fig. 3b,f). This binary image was analysed using the 3D Counter plug-in which quantifies vascular volumes and distribution along the sample’s thickness. As result this plug-in provides a list of all the vessels found along with its corresponding volume in $\upmu \hbox {m}^3$ and the object image that gives the distribution of the identified vessels (Fig. 3c,g). To take into account the volume of the hippocampus in this work we computed the vessel volume fraction (VVF) defined as

2 $$\begin{aligned} VVF=\frac{Vessel\,\,volume}{Hippocampal\,\,volume} \times 1000 \end{aligned}$$

where the hippocampal volume was computed by multiplying the height of the coronal section by the hippocampal surface. The VVF distribution was divided in four different ranges according to the the artery, vein, arteriole, and capillary-venule levels^40^. The ranges for the VVF were: 0–0.0003, 0.0003–0.003, 0.003–1 and > 1. Finally, a single mean VVF value per range, per hippocampus and per mouse was obtained after averaging the VVF distribution from both consecutive confocal images.

### Statistical analysis

From each imaging modality ($\mu$Doppler or SLCM) a single value (quartile cut-off or Mean VVF) was obtained per hippocampus (left or right) belonging to each brain hemisphere. The hippocampi are bilateral structures, with functional lateralization, meaning that left and right hippocampus of the same brain work in complementary manner^41,42^. Moreover, the hippocampal vascular network is not homogeneous in its structure and changes between left and right hippocampus^43,44^. Consequently, to take this variability into account each hippocampus was considered as an independent measurement.

Normality distribution was evaluated using the Shapiro–Wilk test. To assess differences between age, normally distributed parameters (i.e. ranges and quartiles) were compared using the unpaired Student’s *t* test, while non-normal distributions were compared using the Mann–Whitney *U* test. For the evaluation between the different quartiles and ranges within the same age, the one way ANOVA test for normal distributions with Bonferroni’s multiple comparisons test as post-hoc and the Friedman test for non-normal distributions with a two-stage linear step-up procedure of Benjamini, Krieger and Yekutieli as post-hoc were used. All tests underwent two-tailed analysis and the results were considered significant with an alpha level of 0.05. All graphical and statistical analyses were conducted using GraphPad Prism 8 software (GraphPad Prism, RRID:SCR_002798). In this work, there was no excluded data and all outliers were included.

## Results

### Distribution of CBV by $\mu$Doppler analysis

Figure 2a,b show representative coronal $\mu$Dopppler image for a 5-month-old and 21-month-old mouse, respectively. In each $\mu$Doppler image the right hippocampus after segmentation using quartile distribution is highlighted in color. Pixels belonging to the first (Q1), second (Q2), third (Q3) and fourth (Q4) quartiles of the intensity distribution were coloured blue, fuchsia, green and yellow, respectively. As represented in Fig. 2a,b, pixels corresponding to the first quartile Q1 were predominantly located in the center of the hippocampus, while pixels corresponding to Q4 and eventually Q3 were located near the hippocampus boundary. This behaviour was consistent throughout all the experiments. The bar plots in Fig. 2c,d present the average cut-off values of the different quartiles, for 5 and 21-month-old mice, respectively. For a given age, significant differences were found between all quartiles.

### Vascular structure of hippocampus by SLCM analysis

Figures 3a,e show representative tile-scan images for a 5-month-old and 21-month-old mouse, respectively. Vessels marked with IB4 probe appear green. After binarization of the hippocampus (Fig. 3b,f), the mean VVF values were extracted (Fig. 3c,g). The VVF ranges 0–0.0003, 0.0003–0.003, 0.003-1 and >1 were coloured yellow, green, fuchsia and blue, respectively. The bar plots in Fig. 3d,h present the average VVF values for each range for 5 and 21-month-old mice, respectively. For a given age, significant differences were found between ranges.

### CBV and vascular structure vs. age

**Figure 4:**
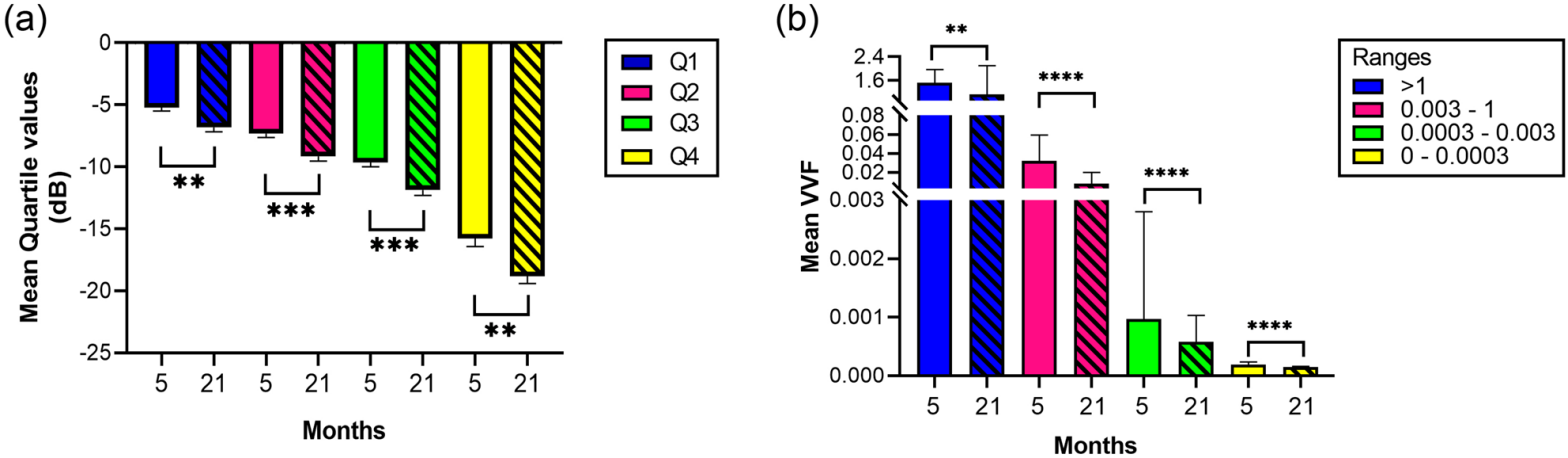
Mean quartile values and VVF for 5- vs. 21-month-old mice. (**a**) Comparison with age of the quartile values obtained by $\mu$Doppler. CBV measured by $\mu$Doppler showed significant higher values for all quartiles when comparing 5- and 21-month-old mice. 5-month-old vs 21-month-old: [Q1]: p = 0.0019, t = 3.537, df = 22; [Q2]: p = 0.0008, t = 3.872, df = 22; [Q3]: p =  0.0005, t = 4.047, df = 22; [Q4]: p = 0.0020, t = 3.504, df = 22. (**b**) Comparison with age of the mean VVF values obtained by SLCM. The VVF values determined by IB4 probing, tile-scan imaging and 3D Counter FIJI plugin, showed significant differences between 5- and 21-month-old mice. 5-month-old vs 21-month-old: [> 1]: p = 0.0023, U = 19,535; [0.003–1], [0.0003–0.003], [0–0.0003]: p < 0.0001, U = 210,691. **p  < 0.0021, ***p < 0.0002, ****p < 0.0001.

Figure 4 compares the average quartile and mean VVF values in terms of age (i.e. 5- vs. 21-month-old mice). We observe that for all quartiles younger mice have significant higher cut-off values (p $\le$ 0.002 for all quartiles in Fig. 4a). Because no significant differences were found in ultrasound attenuation with age ($0.22 \pm 0.08$ dB/cm and $0.21 \pm 0.06$ dB/cm, for young and old mice, respectively), the difference in the quartile cut-off values can be attributed to a decrease in CBV. This is consistent with the results from confocal microscopy (Fig. 4b) where we also find a significant decrease in VVF with age.

### Association between CBV and vascular structure

**Figure 5:**
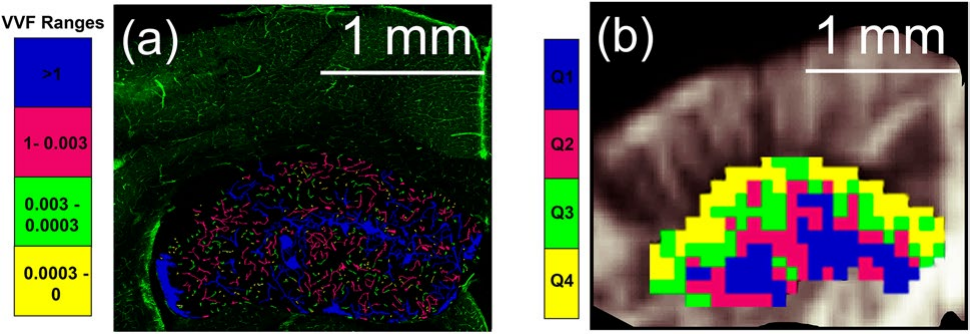
CBV and vascular structure. (**a**) A tile-scan image of the hippocampal endothelial vascular IB4 signal obtained by SLCM (green image) is overlapped with the 3D counter object image showing the identified ranges of volume distribution. Vessels in the 0–0.0003, 0.0003–0.003, 0.003–1 and > 1 range were coloured yellow, green, fuchsia and blue, respectively. (**b**) $\mu$Doppler image overlaid with segmented hippocampus with CBV quartile distribution. Blue, fuchsia, green and yellow corresponds to quartiles Q1, Q2, Q3 and Q4, respectively.

In Fig. 5 SLCM (Fig. 5a) and $\mu$Doppler (Fig. 5b) images corresponding to 5-month-old mice are used to associate CBV regions with its underlying vascular structure. CBV spatial distribution imaged by $\mu$Doppler qualitatively characterizes the functionality of the hippocampal vascular network. As observed in Fig. 5b, high intensity Doppler signals (i.e. Q1 and Q2) are predominantly located in the center region of the hippocampus. We believe this is related to a specific large structure (i.e. VVF ranges > 1 in Fig. 5a) corresponding to the great ventral artery and sulcal vein pathways^45^. Due to the low spatial resolution of $\mu$Doppler, it is highly probable that vessels of different sizes contribute to signals observed in the $\mu$Doppler image.

## Discussion

In this work $\mu$Doppler and SLCM were used to study blood volume and vascular structure in mice hippocampi of different age. The $\mu$Doppler measurements presented in this work integrate vessel volume and blood velocity into one single magnitude proportional to CBV. The goal of introducing a $\mu$Doppler segmentation was: (1) to obtain a set of mice-independent measures that summarizes the CBV distribution across the hippocampus and (2) that these measures can be useful in inter-cohort analysis of different process (e.g. in this work we apply it to study the effect of aging). The segmentation into quartiles was chosen after testing different options: we tested from the median value (i.e. Q2) to a segmentation into deciles. We found that the quartile segmentation allowed us to obtain significant differences between quartiles (Fig. 2c,d) and that this segmentation was able to characterize brain perfusion compartments reflecting vascular physiology^1,3^ (Fig. 5). Moreover, we show that this segmentation was sensitive enough to detect the effect of aging (Fig. 4). The quartile segmentation is a simple and repeatable type of segmentation, which has been corroborated by SLCM (Figs. 3 and 4). However, we do not discard the possibility of other segmentation that may be more suited to study different processes.

Figure 5 illustrates the connection between the quartile segmentation and its underlying vascular structure. High-intensity Doppler signals (i.e. quartiles Q1 and Q2 in Fig. 5b) are predominantly located in the central region of the hippocampus and are most probably linked to the great ventral artery and sulcal vein pathways, highlighted by large VVF ranges (i.e. VVF > 1 and 0.003–1 range in Fig. 5a). Unfortunately, the lack of fiducial markers and the challenges arising from comparing plane slices that can have slightly different alignments and deformations due to experimental manipulation (i.e. brain extraction, fixation and cutting) prevent us from performing a pixel by pixel comparison between both imaging modalities (i.e. a correlative approach). This task is out of the scope of the present work. However, with the help of the Praxinos and Franklin’s mouse brain atlas in stereotaxic coordinates^37^ (i.e. coronal sections corresponding to approximately bregma − 1.58 mm), morphological references (i.e. position and size of central and lateral ventricles) and external markers (position of craniotomy window) we assert that both images in Fig. 5 correspond approximately to the same region of the hippocampus. Moreover, the correspondence between SLCM and $\mu$Doppler segmentation was consistently observed for all mice. Future works could focus on developing a pixel-by-pixel approach with the aid of fiducial markers, morphometric approaches or software to automatically recognize and overlay the combined evidence between the two imaging modalities.

The quartile segmentation method showed to be sensitive enough to find significant differences for all quartiles with age (Fig. 4a). These results are consistent with previously reported results using alternative ultrasonic methods. The work of Li et al.^28^ proposed an ultrafast Doppler method based on 40 MHz ultrasound to study brain vasculature in mice. To this end, Li et al. computed the vascular density defined as the ratio between the number of pixels with Doppler signal and the number of pixels of the ROI (i.e. similar to the VVF used in this work). Experiments in the hippocampus of wild-type mice showed a smaller vascular density with no significant differences for 11-month-old vs. 4-month-old mice (i.e. 7 months of age difference). In our work the age difference between mice groups was 16 month, more than twice the age difference reported in the work of Li et al.^28^. Additionally, the main drawback of any high frequency ultrasound-based imaging modality is its low penetration depth because of ultrasound attenuation. Particularly, in Li et al.^28^ a maximum imaging depth of $\approx$ 3 mm is achieved. The $\mu$Doppler method presented here allows imaging the whole brain and can be easily extended to different regions other than the hippocampus.

Another ultrasonic method to study brain vasculature is Ultrasound Localization Microscopy (ULM). ULM has been capable of detecting vessels of $\approx$ 9 $\upmu$m in diameter and resolving between vessels up to $\approx$ 17 $\upmu$m close^27^. Recent work of Lowerison et al.^46^ used ULM to study microvascular changes with respect to ageing in $\approx$ 7-month-old vs. $\approx$ 27-month-old wild-type mice. In the hippocampus, they found a significant decrease in blood velocity with a significant increase in vascular tortuosity in the aged mice, while no significant differences were found in blood volume and vascularity. In Lowerison et al.^46^ blood volume was estimated using the mean number of microbubbles that entered a particular ROI and brain vascularity was calculated by binarizing the ULM images to determine the percentage of cross-section that was perfused (i.e. similar to the VVF used in this work). Since in the work of Lowerison et al.^46^ aged mice had lower blood velocity, without differences in blood volume and vascularity, one can hypothesize that aged mice would have lower CBV values in agreement with our $\upmu$Doppler results. However, this comparison should be taken carefully because experiments in Lowerison et al.^46^ were conducted at bregma − 3 mm, while ours were conducted at approximately bregma − 1.58 mm.

The major drawback of $\mu$Doppler method used in this work is that a cranial window had to be surgically opened in order to allow undistorted propagation of ultrasound. This kind of invasive procedure should be preferably avoided, especially in prospective longitudinal studies where animals are their own control, e.g. where the same animal is evaluated before and after treatment. Future works should focus in combining transcranial $\mu$Doppler imaging with quartile segmentation. This can be achieved with contrast agents to increase SNR of Doppler signal^47^, by using phase-aberration correction for the skull^48,49^ or skull thinning procedures^50^.

A strong merit of $\mu$Doppler is its capability of performing in vivo imaging and obtaining information of CBV. On the other hand, SLCM allows vascular structural imaging with a very high spatial resolution—almost a factor 100 when compared to $\mu$Doppler—but without CBV information. The incorporation of SLCM coronal brain sections, combined with $\mu$Doppler analysis, enhanced the understanding of the structure of the vascular network as a continent of blood flow. Specifically, it allows us to associate an in vivo coronal CBV image to a post mortem highly resolved vascular structural image obtained with the aid of specific biomarkers for the epithelial membrane of vessels.

In the analyzed hippocampus, the established VVF ranges were in line with the canonical reserve capacity profiles of vein-artery, venule, arteriole, and capillary. The classification of vessel sizes into ranges, revealed significant differences in the mean VVF values between contiguous ranges in both age groups (Fig. 3). With this tool, we were able to assess the impact of ageing at the vascular level by comparing the mean VVF of 5- and 21-month-old mice, demonstrating significant differences in the distribution of volumes in all analyzed ranges (Fig. 4b). The significant differences observed between ranges for total VVF at each age (see Supplementary Fig. S2), were not reflected in either total VVF or VVF between ranges, when comparing both age groups (see Supplementary Figs. S1 and S4). Additionally, the number of vessels also showed significant differences between ranges in both age groups (see Supplementary Fig. S3). As an important corollary, the volume of vessels remains unchanged with age while the number of vessels changes, being greater in the 21-month-old mice. The distribution in ranges of the number of vessels allowed us to recognize that this difference is centred on the smaller vessels (capillaries). The ratio between total VVF per range and the corresponding number of vessels revealed that only the smallest vessel group showed significant differences (see Supplementary Fig. S4). Thus, the lower volume/vessel ratio in the 21-month-old mice could indicate structural deterioration at the capillary level, with an impact on respiratory physiology and gas exchange. This data is specially important because capillaries are part of the neurovascular unit, and may be associated with a specific impairment in vascular perfusion related to impaired neurovascular coupling, as involved in ageing^1–3^. Also, this result is consistent with previous histological studies reporting that ageing is associated with a decrease in microvascular density^51^. Moreover, this is also consistent with trends observed in Li et al.^28^ for approximately the same coronal section. This could be a sufficiently sensitive parameter to assess the temporal changes associated with ageing at the vascular level.

Using the comparative imaging developed in the present work, it is possible to assign to the quartile distribution of CBV determined by $\mu$Doppler, ranges of VVF corresponding to the canonical vascular structure determined by SLCM (Figs. 4 and 5). In the present work, $\mu$Doppler results were cross validated with SLCM. Particularly, vascular structural information (mean VVF distribution) obtained via SLCM supports and cross validates the changes with age in the quartile segmentation introduced for $\mu$Doppler (Fig. 4). Differences in VVF between young and old mice demonstrate a detriment of the vascular network that can be clearly associated with the decrease in $\mu$Doppler intensity, both of which are related to the normal ageing process. The impact of ageing on vascular physiology can thus be comprehensively assessed. The here proposed method is a valuable and robust tool to establish a structural and functional correlate for the study of vascular changes associated with acute (i.e. ischaemia) or chronic (i.e. psychiatric diseases) conditions, as well as in the evaluation of neurodegenerative mechanisms.

## Conclusion

In this work CBV in the hippocampus was quantified by segmenting each $\mu$Doppler image in quartiles of their intensity distribution. The quartile cut-off values are a mouse-independent measure that can be used in robust inter-cohort statistical analyses. Similarly, the vascular structure was characterized by SLCM, obtaining the VVF distribution. Our results showed that high $\mu$Doppler signals are related to specific vessel locations with large VVF. Moreover, significant changes were found in the mean quartile values and vasculature due to ageing. Overall, our approach, which combines high and low spatially resolved imaging techniques, allowed us to associate $\mu$Doppler measurements with the vascular structure. We believe this approach will be effective in studying other acute (e.g. brain injuries) or pathologically (e.g. neurodegeneration) induced changes.

## Supplementary Information

Supplementary Figures.

## Data Availability

The datasets used and analysed during the current study are available from the corresponding authors on reasonable request.
