# Publisher Correction: Intensity distribution segmentation in ultrafast Doppler combined with scanning laser confocal microscopy for assessing vascular changes associated with ageing in murine hippocampi

**DOI:** 10.1038/s41598-022-11822-4

**Published:** 2022-05-10

**Authors:** Maximiliano Anzibar Fialho, Lucia Vázquez Alberdi, Mariana Martínez, Miguel Calero, Jerome Baranger, Mickael Tanter, Juan Pablo Damián, Carlos Negreira, Nicolás Rubido, Alejandra Kun, Javier Brum

**Affiliations:** 1 Laboratorio de Acústica Ultrasonora, Instituto de Física, Facultad de Ciencias, Universidad de La República, 11400 Montevideo, Uruguay; 2 Física No Lineal, Instituto de Física de Facultad de Ciencias, Universidad de La República, 11400 Montevideo, Uruguay; 3 Laboratorio de Biología Celular del Sistema Nervioso Periférico, Departamento de Proteínas y Ácidos Nucleicos, Instituto de Investigaciones Biológicas Clemente Estable, 11600 Montevideo, Uruguay; 4 Chronic Disease Programme (UFIEC), CIEN Foundation, Instituto de Salud Carlos III, CIBERNED and, 28220 Madrid, Spain; 5 Physics for Medicine Paris, Inserm U1273, ESPCI Paris, PSL University, CNRS UMR 8063, 75012 Paris, France; 6 Departamento de Biociencias Veterinarias, Facultad de Veterinaria, Universidad de La República, 13000 Montevideo, Uruguay; 7 Sección Bioquímica, Facultad de Ciencias, Universidad de La República, 11400, Montevideo, Uruguay; 8 Present Address: Institute for Complex Systems and Mathematical Biology, University of Aberdeen, King’s College, Aberdeen, AB24 3UE UK

Correction to: *Scientific Reports* https://doi.org/10.1038/s41598-022-10457-9, published online 26 April 2022

The original version of this Article omitted an affiliation for Alejandra Kun. The correct affiliations are listed below.

Laboratorio de Biología Celular del Sistema Nervioso Periférico, Departamento de Proteínas y Ácidos Nucleicos, Instituto de Investigaciones Biológicas Clemente Estable, 11600, Montevideo, Uruguay

Sección Bioquímica, Facultad de Ciencias, Universidad de la República, 11400, Montevideo, Uruguay

The original Article has been corrected.

