# Supplementary material for: Intensity distribution segmentation in ultrafast Doppler combined with scanning laser confocal microscopy for assessing vascular changes associated with ageing in murine hippocampi
Source: Sci Rep. 2022 Apr 26;12:6784. doi: 10.1038/s41598-022-10457-9 (PMC9042937; doi:10.1038/s41598-022-10457-9)
Supplement: Supplementary file 1 — Supplementary Figures. [file 41598_2022_10457_MOESM1_ESM.pdf]

Supplementary Information

Intensity distribution segmentation in ultrafast Doppler combined with scanning laser confocal microscopy for assessing vascular changes associated with ageing in murine hippocampi

Maximiliano Anzibar Fialho[1,2,+], Lucia Vázquez Alberdi[3,+], Mariana Martínez[3,+], Miguel Calero[4], Jerome Baranger[5], Mickael Tanter[5], Juan Pablo Damián[6], Carlos Negreira[1], Nicolás Rubido[7,2], Alejandra Kun[3,8,*], and Javier Brum[1,]**

[1]Laboratorio de Acústica Ultrasonora, Instituto de Física, Facultad de Ciencias, Universidad de la República, Montevideo, 11400, Uruguay

[2]Física No Lineal, Instituto de Física de Facultad de Ciencias, Universidad de la República, Montevideo, 11400, Uruguay

[3]Laboratorio de Biología Celular del Sistema Nervioso Periférico, Departamento de Proteínas y Ácidos Nucleicos, Instituto de Investigaciones Biológicas Clemente Estable, Montevideo, 11600, Uruguay

[4]Chronic Disease Programme (UFIEC), Instituto de Salud Carlos III, CIBERNED and CIEN Foundation, Madrid, E-28220, Spain

[5]Physics for Medicine Paris, Inserm U1273, ESPCI Paris, PSL University, CNRS UMR 8063, Paris, 75012, France

[6]Departamento de Biociencias Veterinarias, Facultad de Veterinaria, Universidad de la República, Montevideo, 13000, Uruguay

[7]Institute for Complex Systems and Mathematical Biology, University of Aberdeen, King's College, Aberdeen, AB24 3UE, United Kingdom

[8]Sección Bioquímica, Facultad de Ciencias, Universidad de la República, Montevideo, 11400, Uruguay

*akun@fcien.edu.uy **jbrum@fisica.edu.uy

+these authors contributed equally to this work

In this Supplementary Information we present additional magnitudes computed from the vascular volume distribution provided by the 3D Counter plug-in. More precisely, we present the behavior of the Total VVF and number of vessels among ranges and with respect to age. The Total VVF corresponds to the sum of all VVF found in a given range and the number of vessels corresponds to the total number of vessels found in a given range.

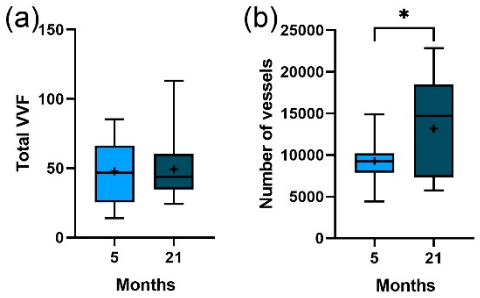

Supplementary Figure S1. Total vessel volume fraction (VVF) and total number of vessels for 5- and 21-month-old mice. (a) Total vessel volume, normalized by the volume of each hippocampus, showed no significant differences between 5- and 21-month-old mice. 5 vs. 21 months: $p = 0.85$, $U = 223$. (b) However, the total number of vessels shows significant differences, being higher in 21-month-old mice. 5 vs. 21 months: $p = 0.0230$, $t = 2.484$, $df = 18$. *$p < 0.05$.

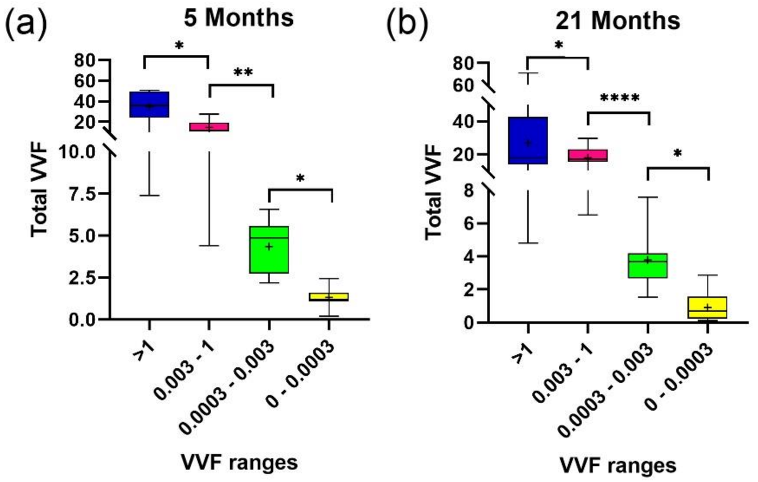

Supplementary Figure S2. Total VVF per range. Total volumes, normalized by the volume of each hippocampus, were separated in four ranges. Comparison of contiguous ranges showed significant differences within each age group. (a) For 5-month-old mice: $\chi^2(4) = 30$, [0-0.0003 vs. 0.0003-0.003]: p = 0.0470; [0.0003-0.003 vs. 0.003-1]: p = 0.0067; [0.003-1 vs. >1]: p = 0.0201. (b) For 21-month-old mice: $\chi^2(4) = 30$, [0-0.0003 vs. 0.0003-0.003]: p = 0.0239; [0.0003-0.003 vs. 0.003-1]: p < 0.0001; [0.003-1 vs. >1]: p = 0.0252. *p < 0.05, **p < 0.0021, ****p < 0.0001.

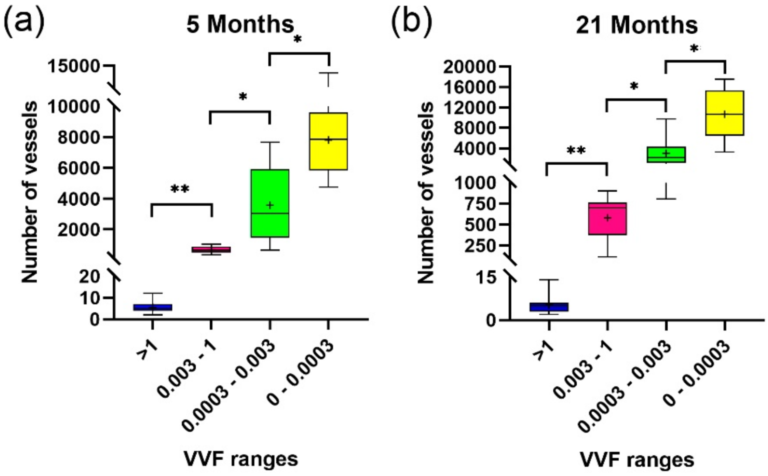

Supplementary Figure S3. Number of vessels per range. Number of vessels for each hippocampus were separated in four ranges. (a) For 5-months-old, comparison of contiguous ranges showed significant differences within each age group: $F_{(3,36)} = 11.25$, [0-0.0003 vs. 0.0003-0.003]: p = 0.048; [0.0003-0.003 vs. 0.003-1]: p = 0.0001; [0.003-1 vs. >1]: p = 0.0018. (b) For 21-month-old mice, comparison of contiguous ranges showed significant differences within each age group: $F_{(3,36)} = 18.04$, [0-0.0003 vs. 0.0003-0.003]: p = 0.0304; [0.0003-0.003 vs. 0.003-1]: p = 0.0422; [0.003-1 vs. >1]: p = 0.002. *p < 0.05, **p < 0.0021.

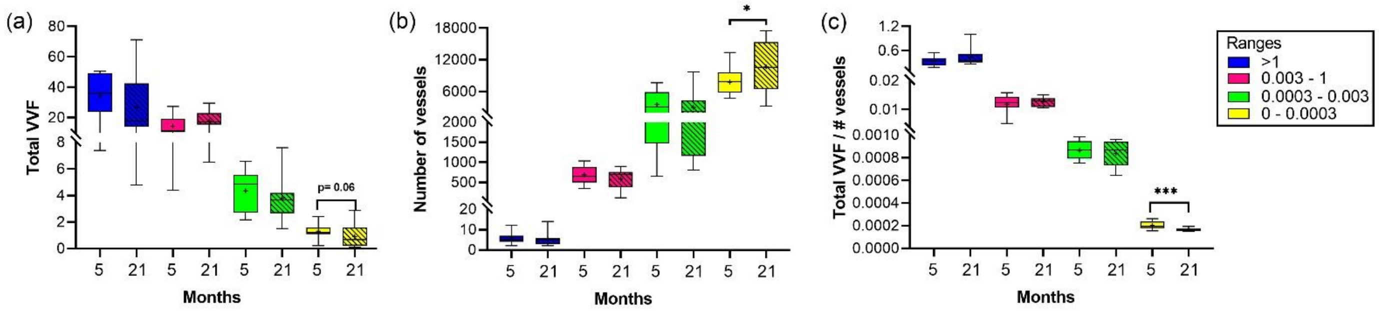

Supplementary Figure S4. Comparative quantification of vascular brain network between 5- and 21-month-old mice. (a) Total VVF showed no significant differences for any of the volume ranges analyzed, although there was a trend in the range corresponding to vessels of smaller volume. 5 vs. 21 months: [>1]: p = 0.1308, U = 85; [0.003-1]: p = 0.7050, t = 0.4239, df = 18; [0.0003-0.003]: p = 0.2861, U = 82; [0-0.0003]: p = 0.0626, t = 1.832, df = 18. (b) Number of vessels per range showed significant differences only in the smaller vessels, with a significant increase in the vessels of older mice 5 vs. 21 months: [>1]: p = 0.475, U=68; [0.003-1]: p = 0.2022, U=78; [0.0003-0.003]: p = 0.3233, U = 80; [0-0.0003]: p = 0.0457, U = 42. (c) Total VVF per range divided by the number of vessels in this range. This ratio showed a significant difference for the lowest volume range, indicating that the young mice (5-month-old) had a highly significant mean, relative to the 21-month-old mice. 5 vs. 21 months: [>1]: p = 0.1230, U = 29; [0.003-1]: p = 0.1655, U = 31; [0.0003-0.003]: p = 0.3623, t = 0.9346, df = 18; [0-0.0003]: p = 0.0006, t = 3.821, df = 18. *p < 0.05, ***p < 0.0002.
